# Expanding the Genotypic Spectrum of POMGNT1‐Related Muscle‐Eye‐Brain Disease: A Case Report

**DOI:** 10.1002/mgg3.70256

**Published:** 2026-08-07

**Authors:** Evripidis Pityrigkas, Vasiliki Poulidou, Stefania Kalampokini, Eleni Liouta, Georgia Pepe, Martha Spilioti, Vasilios K. Kimiskidis

**Affiliations:** ^1^ First Department of Neurology, AHEPA University Hospital Aristotle University of Thessaloniki Thessaloniki Greece; ^2^ Genekor Medical S.A. Athens Greece

**Keywords:** developmental and epileptic encephalopathy, dystroglycanopathy, muscle‐eye‐brain disease, *POMGNT1*

## Abstract

**Background:**

Muscle‐Eye‐Brain disease (MEB) is a rare autosomal recessive dystroglycanopathy caused by defective glycosylation of α‐dystroglycan, leading to a multisystem disorder involving the central nervous system, skeletal muscle, and eyes. It represents an important cause of developmental and epileptic encephalopathy, with variable clinical severity and genetic heterogeneity, most commonly associated with variants in the *POMGNT1* gene.

**Methods:**

Clinical, neurophysiological, neuroimaging, histopathological, and genetic investigations were performed, including targeted next‐generation sequencing for congenital muscular dystrophy‐associated genes.

**Results:**

The patient presented with early‐onset hypotonia, global developmental delay, progressive motor regression, intellectual disability, and ocular abnormalities. Epilepsy onset occurred in childhood and evolved into drug‐resistant epilepsy with status epilepticus in adulthood. Brain MRI revealed lissencephaly, ventriculomegaly, and brainstem and cerebellar hypoplasia. EEG demonstrated features consistent with epileptic encephalopathy. Genetic testing identified compound heterozygosity for the *POMGNT1* variants c.1282C>T, p.(Gln428*) and c.793C>T, p.(Arg265Cys). Seizure control was eventually achieved with polytherapy including four antiseizure medications.

**Conclusion:**

This case expands the genotypic and phenotypic spectrum of *POMGNT1*‐related MEB, highlighting the potential for severe epilepsy and status epilepticus in adulthood. It also underscores the importance of long‐term clinical follow‐up and suggests that aggressive antiseizure treatment may achieve seizure control even in advanced stages of the disease.

## Graphical abstract

Compound heterozygous variants in the POMGNT1 gene expand the genotypic spectrum of Muscle‐Eye‐Brain disease, highlighting severe epilepsy with status epilepticus. Despite long disease duration, seizure freedom was achieved with intensive antiseizure polytherapy, underscoring the importance of continued therapeutic optimization in dystroglycanopathies.

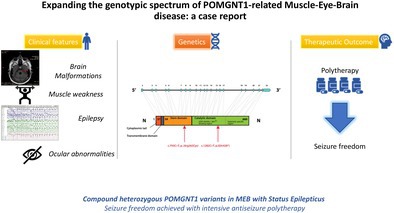

## 1 Introduction

Muscle‐eye‐brain disease (MEB) is an uncommon autosomal recessive congenital muscular dystrophy classified among the dystroglycanopathies (Kang et al. 2015). The disorder arises from impaired glycosylation of α‐dystroglycan (α‐DG), a pivotal component of the dystrophin‐glycoprotein complex that anchors the cytoskeleton of muscle and neuronal cells to the extracellular matrix (Barresi and Campbell 2006; Dobson et al. 2013; Kanagawa 2021). Within the central nervous system (CNS), α‐DG is predominantly localized to astrocytic processes that form the glia limitans, whereas in the retina it is expressed on photoreceptor cells. Deficient glycosylation compromises α‐DG's ability to bind extracellular ligands such as laminin‐2, agrin, and neurexin, weakening the structural integrity of both muscle and neural tissues (Barresi and Campbell 2006; Ross 2002). This disruption underlies the multisystemic manifestations of MEB, which include neonatal hypotonia, delayed acquisition of motor milestones, cognitive impairment, progressive muscular weakness, joint contractures, ocular defects (including myopia, strabismus, retinal abnormalities, and optic atrophy), and epilepsy (Kang et al. 2015; Song et al. 2021). Seizures in MEB frequently contribute to a developmental and epileptic encephalopathy, with a broad spectrum of types and severity.

Variants in *POMGNT1* (Protein O‐linked mannose β1,2‐N‐acetylglucosaminyltransferase 1) are the most commonly reported genetic cause, typically occurring in homozygous or compound heterozygous states (Taniguchi et al. 2003; Yoshida et al. 2001). The gene encodes a type II transmembrane glycosyltransferase responsible for O‐mannosylation of α‐DG, a modification essential for proper CNS and muscle function (Dobson et al. 2013). Loss‐of‐function variants in *POMGNT1* abolish enzymatic activity, resulting in defective α‐DG glycosylation and the characteristic clinical features of MEB (Godfrey et al. 2007; Manya et al. 2003).

Herein, we present a case of an adult patient with MEB due to compound heterozygous *POMGNT1* variants, who developed drug‐resistant epilepsy complicated by status epilepticus, highlighting the long‐term neurological evolution and management challenges of this rare disorder.

## 2 Clinical Report

A 32‐year‐old man presented to the emergency department with status epilepticus (SE), characterized by recurrent generalized tonic–clonic seizures without recovery of consciousness between events, despite administration of rectal diazepam as rescue therapy.

His past medical history was notable for neonatal hypotonia and global neurodevelopmental delay. He achieved sitting and first words at 4 years of age and initiated walking at 6 years. Subsequently, he experienced developmental regression, with progressive loss of speech and independent walking, ultimately becoming wheelchair‐dependent. At the age of 9 years he developed epilepsy, manifesting as absence, focal, and generalized tonic seizures, which were partially controlled with valproic acid. Ophthalmological history included severe myopia and strabismus; electroretinography demonstrated a markedly reduced b‐wave. Family history was unremarkable.

**FIGURE 1:**
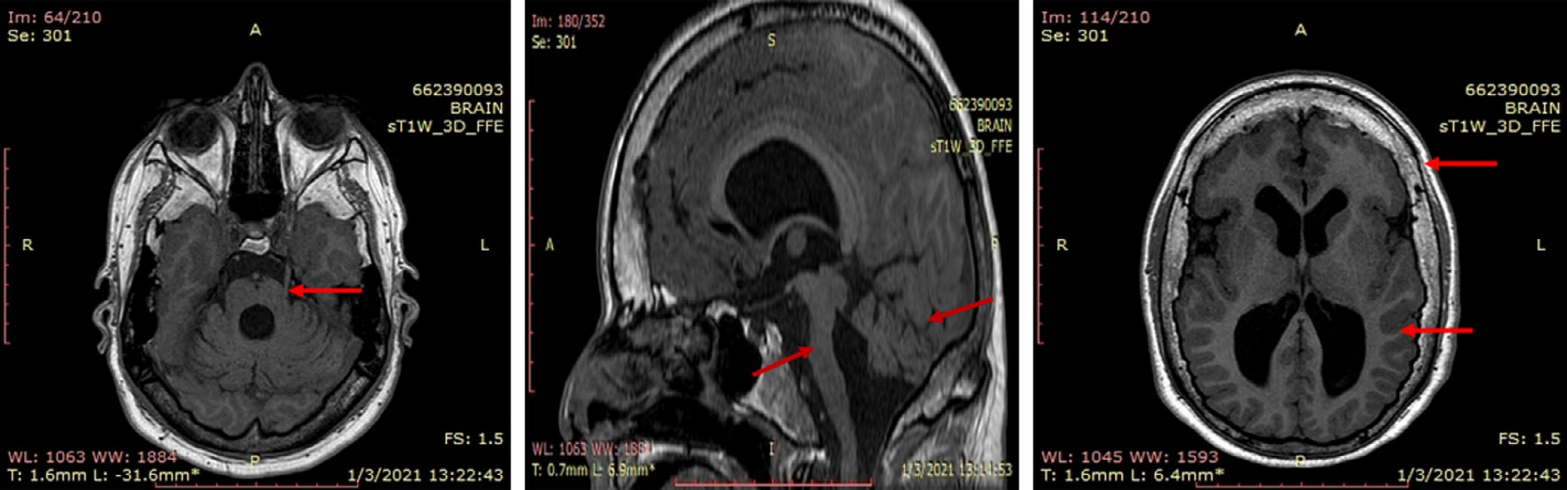
T1‐weighted MRI imaging of the brain showing brainstem and cerebellar hypoplasia, ventricular enlargement and lissencephaly predominantly involving the frontal lobes (red arrows).

Neurological examination revealed flaccid weakness of the upper limbs and spastic paraparesis with brisk deep tendon reflexes, clonus, and joint contractures in the lower limbs. Ophthalmological evaluation showed bilateral optic atrophy. There were no facial dysmorphic features. Brain magnetic resonance imaging (MRI) demonstrated predominantly frontal lissencephaly, hypoplasia of the corpus callosum, brainstem, and cerebellum and ventriculomegaly (Figure 1).

Following resolution of SE, the patient exhibited daily focal seizures with impaired awareness, associated with tachycardia, respiratory distress, upward eye deviation, and myoclonic jerks of the upper limbs, each lasting less than 1 min.

**FIGURE 2:**
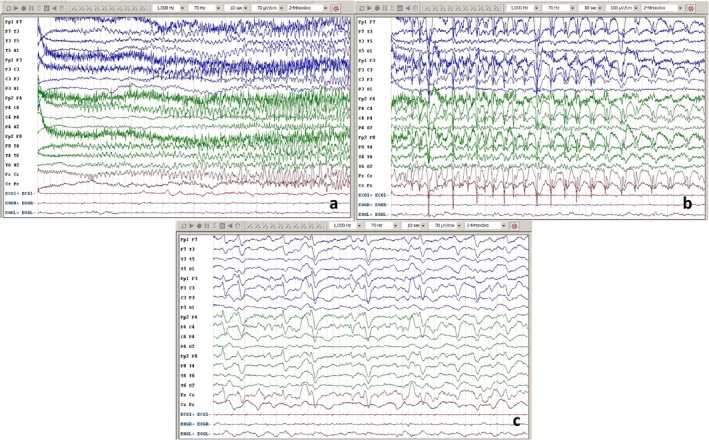
(a) Ictal EEG revealing fast activities over the frontocentral areas on both hemispheres, intermixed with muscle artifacts, (b) ictal EEG revealing sharp wave complexes at 2.5 Hz, most prominent over frontocentral areas and (c) Interictal EEG consistent with epileptic encephalopathy, showing periodic discharges of sharp‐slow complexes bilateral, prominent at frontocentral areas.

Ictal EEG showed fast activity (25–30 Hz) over the frontal regions, evolving into sharply contoured theta activity and 2.5‐Hz spike–wave or sharp‐slow wave complexes (Figure 2a,b). Interictal EEG was consistent with epileptic encephalopathy, displaying diffuse theta‐delta slowing and bilateral periodic sharp‐slow wave discharges, predominantly over the frontocentral regions (Figure 2c).

Seizure control was achieved with a combination of valproic acid (2500 mg/day), brivaracetam (200 mg/day), lacosamide (300 mg/day), and zonisamide (100 mg/day), resulting in complete seizure freedom.

Laboratory evaluation revealed persistently elevated serum creatine kinase levels, also documented outside seizure episodes. Previous metabolic investigations, including plasma amino acids and urinary organic acids, were normal. Needle electromyography demonstrated a myopathic pattern. A muscle biopsy with immunohistochemical studies, performed approximately 5 years earlier, revealed myopathic changes and reduced expression of α‐dystroglycan, laminin α2, and laminin β2, thereby establishing the diagnosis of ΜΕΒ disease.

**FIGURE 3:**
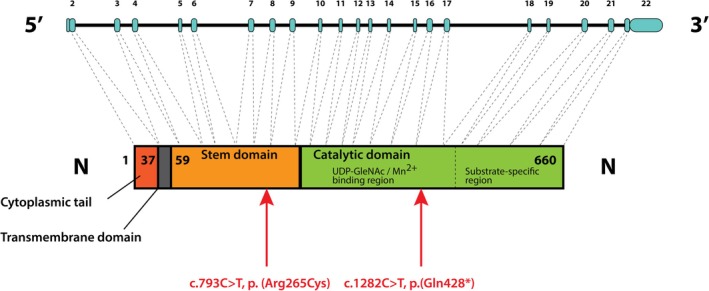
*POMGNT1* gene and the corresponding protein with its main domains; the variants found in the present case are shown with red arrows.

Based on the patient's phenotype, a gene panel targeting congenital muscular dystrophies and dystroglycanopathies was performed using next‐generation sequencing (NGS), which revealed compound heterozygosity for the variants NM_017739.4:c.1282C>T, p.(Gln428*) and NM_017739.4:c.793C>T, p.(Arg265Cys) in exons 15 and 9 of the *POMGNT1* gene, respectively (Figure 3).

The nonsense variant c.1282C>T, p.(Gln428*) is a previously reported pathogenic variant (ClinVar VCV000970328.8) that introduces a premature stop codon at position 428, resulting in a truncated, non‐functional protein. Loss‐of‐function variants in *POMGNT1* have been previously reported as pathogenic in multiple individuals with ΜΕΒ disease, both in homozygosity and compound heterozygosity (Borisovna et al. 2019; Endo et al. 2010; Manya et al. 2003).

The missense variant c.793C>T, p.(Arg265Cys) results in the replacement of a positively charged arginine with a polar cysteine residue at codon 265. This residue lies outside Pfam‐defined catalytic domains (e.g., GNT‐I), but falls within the N‐terminal region previously referred to as the “stem domain,” which has been implicated in glycan recognition and enzyme interactions (Kuwabara et al. 2016). The variant is described in population databases (rs774752168), with a global allele frequency < 0.01% in gnomAD v4.1.0 (GRCh38; accessed February 2026), and has been submitted to ClinVar as a variant of uncertain significance (Variation ID: 1209698). In silico predictive tools support a deleterious effect (REVEL score: 0.78). Based on ACMG guidelines (Richards et al. 2015), the variant was initially classified as a variant of uncertain significance (PM2, PM5, PP3). Representative chromatograms confirmed the presence of both variants in the patient, demonstrated maternal inheritance of c.1282C>T and showed absence of the c.793C>T variant in both parents (Figure S1).

## 3 Discussion

In this case, the patient has one clearly pathogenic variant p(.Gln428*) and the variant p(.Arg265Cys) in the *POMGNT1* gene. The patient's mother, who is clinically unaffected, was found to be heterozygous for the pathogenic nonsense variant p.(Gln428*), consistent with the autosomal recessive inheritance pattern observed in dystroglycanopathies. The p.(Arg265Cys) variant was initially classified as a variant of uncertain significance based on ACMG criteria PM2 (very low population frequency), PM5 (novel missense change at a residue with a known pathogenic missense variant), and PP3 (computational predictions indicating a deleterious effect). Segregation analysis revealed that this variant was not detected in either parent, raising the possibility of a de novo occurrence; however, paternity was not confirmed, and therefore a true de novo origin cannot be definitively established, representing a limitation of the present study. Importantly, the p.(Arg265Cys) variant affects the same amino acid residue as previously reported pathogenic substitutions, including p.(Arg265His) and p.(Arg265Pro), which have been identified in multiple individuals with MEB in compound heterozygosity (Fu et al. 2017; Song et al. 2021; Vervoort et al. 2004). This recurrence of distinct pathogenic substitutions affecting Arg265 highlights the functional importance of this residue and raises the possibility that it represents a mutational hotspot within the stem domain of *POMGNT1*. In addition, multiple sequence alignment demonstrated strong evolutionary conservation of the Arg265 residue across species, highlighting its functional importance (Figure S2). These observations further support the interpretation that the p.(Arg265Cys) variant is likely disease‐causing in this genetic context. Additional functional studies and/or identification of the variant in unrelated affected individuals would help to clarify its pathogenic significance.

The *POMGΝT1* gene comprises 22 exons and encodes a protein of 660 amino acids which is divided into four domains: a cytoplasmic tail (Met1‐Arg37), a transmembrane domain (Phe38‐Ile58), a stem domain (Leu59‐Leu300), the catalytic domain consisting of the UDP‐GlcNAc and Mn^2+^ binding regions (Asn301‐Leu530), and the substrate‐specific region (Arg531‐Thr660) (Yoshida et al. 2001). In our patient the variant c.1282C>T, p.(Gln428*) is found in the catalytic domain of the protein, leading to a stop codon and premature ending of translation, that is, a non‐functional protein, while the variant c.793C>T, p. (Arg265Cys) is found in the stem domain. The stem domain recognizes the β‐linked GlcNAc of O‐mannosyl glycan, an enzymatic product of POMGnT1 and this interaction may recruit other enzymes that interact with POMGnT1, for example, fukutin, which is required for further modification of the glycan (Kuwabara et al. 2016). Indeed, all described *POMGNT1* variants lead to partial or complete loss‐of‐function of enzymatic activity of the POMGnT1 protein, which is responsible for the modification of a‐dystroglycan (Godfrey et al. 2007; Manya et al. 2003).

Epilepsy, which may be drug‐resistant, represents a frequent and clinically significant feature among patients with MEB disease (Song et al. 2021; Yang et al. 2022). A broad spectrum of seizure types has been reported in MEB, with absences being the most common, followed by generalized tonic–clonic and focal seizures (Kalampokini et al. 2026). EEG findings in affected individuals typically include an abnormal background characterized by low‐amplitude, slow, and disorganized activity with prominent θ and δ waves (Yang et al. 2022). These EEG abnormalities are generally concordant with neuroimaging findings, most notably type II lissencephaly, which could contribute to epileptogenic network formation in dystroglycanopathies (Yang et al. 2022). In line with these electroclinical and radiological correlations, in the present case, epileptic discharges were predominantly recorded over the frontocentral regions, closely corresponding to the distribution of lissencephaly on brain MRI. A more refined characterization of electroclinical patterns in MEB may facilitate earlier recognition of epilepsy, improve diagnostic accuracy, and inform individualized management strategies in this rare and heterogeneous disorder.

In conclusion, in this study we identified a rare *POMGNT1* missense variant affecting a residue previously implicated in MEB disease, which, in combination with a pathogenic loss‐of‐function allele, is likely to contribute to the observed phenotype. This case expands the genotypic spectrum of *POMGNT1*‐related MEB, emphasizing that severe epilepsy and status epilepticus may occur in adulthood and may still respond to optimized antiseizure polytherapy.

## Author Contributions

Data collection and conceptualization: E.P., V.P., S.K. and E.L. Manuscript writing – original draft: E.P., V.P., S.K. and E.L. Data analysis and interpretation: G.P. Supervision: M.S. and V.K.K. Manuscript review and editing: all authors.

## Funding

The authors did not receive any specific funding for this study.

## Consent

The patient's relatives gave written consent for publication of the case.

## Conflicts of Interest

The authors declare no conflicts of interest.

## Supporting information


**Figure S1:** Representative chromatograms from a family trio showing the c.793C>T (exon 9) and c.1282C>T (exon 15) variants in the *POMGNT1* gene.
**Figure S2:** Sequence alignment of POMGNT1 from different species depicting that the Arg265 residue is highly conserved.

## Data Availability

The data that support the findings of this study are available from the corresponding author upon reasonable request.
