# Supplementary material for: Expanding the Genotypic Spectrum of POMGNT1‐Related Muscle‐Eye‐Brain Disease: A Case Report
Source: Mol Genet Genomic Med. 2026 Aug 7;14(8):e70256. doi: 10.1002/mgg3.70256 (PMC13451836; doi:10.1002/mgg3.70256)
Supplement: Supplementary file 1 [file MGG3-14-e70256-s001.docx]

**Supplementary figure 1.** Representative chromatograms from a family trio showing the c.793C>T (exon 9) and c.1282C>T (exon 15) variants in the *POMGNT1* gene.

c.1282C>T

c.793C>T

a) Patient’s chromatograms showing both c.793C>T and c.1282C>T variants in heterozygosity.

c.793C>T

b) Father: Absence of the c.793C>T variant, indicating a **de novo** origin.

c.1282C>T

c) Mother: Presence of the pathogenic c.1282C>T variant

**Supplementary figure 2.** Sequence alignment of POMGNT1 from different species depicting that the Arg265 residue is highly conserved.
